# Debt, sleep deprivation and psychological distress among online ride-hailing drivers: evidence from China

**DOI:** 10.1136/gpsych-2023-101332

**Published:** 2024-03-14

**Authors:** Wanjie Tang, Jingyue Chen, Simiao Wang, Xianglan Jiang, Yi Lu, Siqi Wu, Luyu Yang, Meng Tian, Han Zhang, Yinan Zhang, Jiuping Xu, Zeyuan Sun

**Affiliations:** 1 Mental Health Centre, Sichuan University, Chengdu, Sichuan, China; 2 Department of Child & Adolescent Psychiatry, King's College London Institute of Psychiatry Psychology & Neuroscience, London, UK; 3 Sichuan University Business School, Chengdu, Sichuan, China; 4 Department of Psychology, King's College London Institute of Psychiatry Psychology & Neuroscience, London, UK; 5 Sichuan University West China School of Medicine, Chengdu, Sichuan, China; 6 The University of Edinburgh School of Health in Social Science, Edinburgh, Edinburgh, UK; 7 Department of Psychology, University of Warwick, Coventry, UK; 8 Division of Psychiatry, University College London Faculty of Brain Sciences, London, UK; 9 Wuxi Mental Health Center, Nanjing Medical University, Wuxi, Jiangsu, China; 10 King's College London Centre for the Developing Brain, London, UK

**Keywords:** Cross-Sectional Studies, Family Conflict, Health Behavior, Mental Health, Psychology, Social

**To the editor:**

An online ride-hailing driver (ORHD) refers to a driver who takes orders and provides rental car services to passengers via an online service platform.^1^ ORHD plays a significant role in the urban transport system worldwide, operating through many platforms. According to official data from the Chinese Ministry of Transport, a total of 1.6 million vehicle transport permits were issued by the end of March 2022. Moreover, by the end of 2021, the number of Chinese online taxi service users has reached 453 million.^2^

ORHDs are characterised by lower levels of education and savings, and most of them are married men.^3^ Motivated by the commission and incentive policies of the online platform, ORHDs tend to work long hours and take few breaks, leading to sleep deprivation.^3 4^ ORHDs face many challenges, such as market competition and operating costs.^5^ Research has identified specific mental health issues prevalent among drivers, such as stress and anxiety,^6^ which may affect their driving quality, job satisfaction and overall well-being.^7^

Many ORHDs start this job due to the burden of debt.^8^ Previous research found that household financial debt can impact physical health by mediating healthcare behaviours and mental health. These associations are more pronounced in individuals who are middle-aged, married and low-income,^9^ which overlaps with the characteristics of ORHDs. Sleep is also associated with the mental health of taxi drivers. In the USA, 52% of drivers reported insufficient sleep during the workweek, and this figure increased due to the deterioration of their financial situation.^10^

The current study aims to: (1) examine mental health issues among ORHDs in China; (2) investigate the association between sleep and mental health among ORHDs; and (3) explore the association between debt, sleep and mental health among ORHDs. To our knowledge, this is the first study providing a comprehensive picture of sleep-related problems among ORHDs.

## Study design and participants

This is a cross-sectional study. A convenience sample of 524 full-time ORHDs from Chengdu, Wuhan and Shenzhen were recruited from ORHD waiting areas at airports, electric charging posts and petrol stations. These locations were identified through communication with ORHD platforms and interviews with drivers to maximise sample representativeness. From April 2021 to January 2022, the research team conducted a survey using the Wenjuanxing platform (www.wjx.cn/). Answers were quality-controlled before submission. All data are considered valid (online supplemental file 1).

10.1136/gpsych-2023-101332.supp1 Supplementary data

## Measures

The design of the questionnaire referred to the previous literature,^11^ and the final version was approved by relevant experts.

## Insomnia severity index

The insomnia severity index (ISI) is a well-validated self-report questionnaire that assesses the perceived severity of sleep deprivation.^12^ A 5-point Likert scale (0–4) is used to score each item. The ISI has a total score range of 0–28, with higher scores indicating more severe sleep deprivation. The reported Cronbach’s alphas ranged from 0.65 to 0.92, while the pooled Cronbach’s alpha based on 29 688 participants was 0.83.^13^

## Kessler-6

Kessler-6 (K-6) is a reliable self-report tool for measuring general psychological distress (Cronbach’s alpha range from 0.81 to 0.94).^14^ It has six items that assess the frequency of emotions including anxiety, helplessness, restlessness and depression over the last month. Each item is scored using a 5-point Likert scale (0–4), with higher scores indicating higher degrees of psychological discomfort. The K-6 score is included as a continuous variable in the study.

## Work-family conflict

The Work-Family Conflict Scale (WFCS) is a validated self-report questionnaire (Cronbach’s alpha=0.899) that evaluates the extent to which work-related obligations interfere with family responsibilities.^15^ It comprises 12 items, with each subscale measured by 3 items scored on a 5-point Likert-type scale (1–5), with higher scores indicating greater conflict. We included the WFCS score as a continuous variable in the study.

In addition to the measures above, we also collected demographic information including age, gender, education level, length of service and car ownership. Sleep-related data, such as sleep duration and nap habits, and debt status were also collected.

Statistical analyses were performed using R V.4.2.1 and RStudio V.1.4.1717. Independent sample t-tests and χ^2^ tests were used to probe differences between groups. The imputation of missing data was conducted using the multivariate imputation (mice package of R). Separate linear regression models were used to test for the significance of sleep deprivation severity and debt status (debt-free vs in-debt) on psychological distress for ORHD. Using likelihood ratio F-tests, the best-fitting model was compared with another model that considered the interaction between debt status, work-family conflict and sleep deprivation severity. Simple slope analyses were performed in the event of a significant interaction to determine the association’s magnitude.

## Data analysis

The age of the participants ranged from 18 to 60 years, with a median age of 38 (SD=8.74) years. 60.50% of the participants reported having debt, while only 30.34% owned the car they used to provide the rental car service. 22.14% of the participants reported sleeping less than 6 hours per day, and 35.31% did not have a nap or had a nap duration of less than 10 min per day (online supplemental table 1).

Online supplemental table 2 demonstrates the difference in sleeping patterns and mental health between the debt-free group and the in-debt group. The participants in debt had significantly poorer sleep quality (t=6.27, p<0.001), higher levels of psychological stress (t=7.02, p<0.001) and more pronounced family-work conflicts (t=5.58, p<0.001). Univariate linear regressions were conducted to further explore the association between sleep deprivation and psychological distress.

A model comparison using the likelihood ratio F-test demonstrated that the model predicting psychological distress from sleep deprivation (with covariates of age, gender, education level, length of service, debt status, family-work conflict, sleep duration and nap duration) was significantly improved by the inclusion of interaction between debt status, sleep deprivation severity and family-work conflict (F=6.82, p<0.001). The result of the model is presented in table 1 (also in online supplemental figures 2 and 3).

**Table 1:** Results of univariate follow-up analyses regressing sleep deprivation and psychological distress

	Estimate (95% CI)	T value	P value
Debt status	−5.21 (−9.82 to −0.60)	−2.22	**0.027**
Age	−0.19 (−0.11 to −0.02)	−3.04	**0.002**
Gender	1.02 (−0.10 to 2.14)	1.79	0.073
Education	0.69 (−0.02 to 1.39)	1.92	0.056
Sleep deprivation severity	−0.19 (−0.50 to 0.11)	−1.24	0.217
Sleep duration	0.36 (−0.22 to 0.94)	1.22	0.222
Nap duration	−0.38 (−0.79 to 0.04)	−1.76	0.079
Length of service	0.84 (0.32 to 1.35)	3.16	**0.002**
Family-work conflict	−0.04 (−0.17 to 0.09)	−0.58	0.563
Sleep deprivation severity × family-work conflict	0.02 (0.01 to 0.03)	3.99	**<0.001**
Sleep deprivation severity × debt status	0.48 (0.09 to 0.86)	2.43	**0.015**
Debt status × family-work conflict	0.20 (0.03 to 0.38)	2.27	**0.024**
Sleep deprivation severity × family-work conflict × debt status	−0.01 (−0.02 to -0.00)	−2.27	**0.024**

The reference group of debt status is debt-free; the reference group of gender is male.

CI, confidence interval.

## Discussion

This study examined the association between sleep deprivation and psychological stress among ORHDs, considering a wide range of work-related and socioeconomic factors. We found that ORHDs with debt and more severe family-work conflict are more likely to suffer from psychological stress due to sleep deprivation. These findings are in line with previous works in China, Iran and Brazil,^16–18^ strengthening the argument that sleep deprivation is a significant risk factor for mental health among ORHDs worldwide.

Age is strongly associated with the psychological distress experienced by ORHDs. While limited research has been conducted among ORHDs, age is commonly linked to behavioural intention and driving behaviour, with older drivers being more likely to have lower intentions and frequencies of unsafe driving behaviours.^5^ Younger ORHDs often work longer hours and take fewer breaks compared with older ORHDs.^19^ This observation may partially explain the findings of this study that older drivers may suffer less from the associations with sleep deprivation on psychological stress due to safer driving behaviours and shorter working schedules in terms of distance and time spent driving.

This study discovered that longer lengths of service may increase the risk of psychological distress among ORHDs. Another study conducted in China found that older drivers and those with more experience and activity are more susceptible to the effects of the increasingly competitive working environment, resulting in income reduction.^20^

A strong relationship between financial difficulties and adverse mental health outcomes has been extensively reported in the literature.^21^ Professional drivers are believed to face elevated stress levels and a heightened likelihood of encountering financial challenges.^22^ This research also found strong associations between debt, sleep deprivation and psychological distress. Despite the combined effect correlation of debt on the sleep deprivation-psychological distress association that exacerbates the association between sleep deprivation and psychological stress (online supplemental figure 2), the effect role of debt is complicated in this case. Its primary effect may mitigate the impact of sleep deprivation on psychological stress, while its interaction effect with family-work conflict and sleep deprivation could amplify the effect of sleep deprivation on psychological stress (table 1).

Our study has several strengths, including a representative sample and comprehensive consideration of many socioeconomic factors compared with previous literature. With a robust design and multiple dimensions of the data, we uncovered correlations between several work-related factors and mental health outcomes, which provide a fresh perspective for the rapidly expanding ORHD industry.

This study has some limitations. First, the nature of the cross-sectional study limited our ability to make causal inferences about our results. Prior studies also noted that sleep deprivation and debt may have a long-term and interactive correlation.^18 23 24^ Therefore, our study may not fully elucidate the dynamic correlations between debt, sleep deprivation and mental health outcomes. Second, our sample size is relatively small for such a large occupation population in China. According to the latest report, there were 5.09 million ORHDs by the end of 2022.^2 25^ Our study is also limited by the non-random sampling method, so our findings based on a comparatively small sample, though from three major cities, may not be generalisable to other regions in China and other countries. Third, the associations between debt, sleep deprivation and psychological distress may require extra caution in interpretation due to the diverse socioeconomic statuses of the participants and varying living expenses across the three cities where our study was conducted. The inconsistency compared with previous studies could also result from insufficient information on potential confounders, such as the specific amount of debt, shift work status, family income and marital status, which were not covered in the questionnaire. Fourth, assessments relied on self-report scales and questionnaires, which may introduce measurement bias. Finally, as our findings are based on a sample from China, their generalisability to other countries may be limited due to differences in working circumstances and regional legislation.

Our findings hold significant implications for the well-being of ORHD and the general public. The association between insufficient sleep and safety hazards is a critical concern, as sleep-deprived drivers are at an increased risk of accidents due to impaired attention and slower reaction times. The insidious nature of sleep deprivation as a potential catalyst for psychological distress underscores the need to recognise it as a crucial public mental health concern. Furthermore, the escalating psychological burden experienced by drivers could potentially impair their quality of life and well-being. The elevated stress levels may further precipitate a ripple impact, negatively influencing their capacity to provide reliable ride-hailing services, and even compromising the safety of passengers. Given the large and growing population of ORHDs and their users, this could pose a potentially significant public health issue. Addressing these multifaceted challenges demands a comprehensive approach. Strategies such as enhancing income by reducing platform commissions can alleviate financial strain and thereby reduce stress levels among drivers. The platform and the relevant authorities should also enforce stricter restrictions on working overtime to prevent fatigue driving. Moreover, the introduction of national policies to safeguard the rights and interests of ORHDs could mitigate their financial vulnerabilities and ensure fair treatment within the broader framework of labour rights. Future research should focus on distinguishing the role of socioeconomic status (eg, debt status and family-work conflicts) from causal effects through longitudinal studies with larger samples.

## Conclusion

This study demonstrates that ORHDs experiencing sleep deprivation are at a higher risk of experiencing higher levels of psychological distress. Moreover, family-work conflict, as well as debt, could amplify the correlation, leading to more severe mental health consequences.
