# Supplementary material for: Debt, sleep deprivation and psychological distress among online ride-hailing drivers: evidence from China
Source: Gen Psychiatr. 2024 Mar 14;37(2):e101332. doi: 10.1136/gpsych-2023-101332 (PMC10941107; doi:10.1136/gpsych-2023-101332)
Supplement: Supplementary data [file gpsych-2023-101332supp001.pdf]

Supplemental table 1: Sociodemographic and sleep-related characteristics of respondents (N=524)

Characteristics	Total sample
**Age, Median (range)**	38.0 (18, 60)
**Male, n (%)**	464 (88.54)
**Education, n (%)**	
Compulsory education and below	386 (73.80)
Undergraduate	114 (21.80)
Postgraduate and above	23 (4.40)
**Debt status, n (%)**	
Debt-free	207 (39.50)
In debt	317 (60.50)
**Length of service, n (%)**	
2 years or less	153 (29.20)
2–5 years	260 (49.62)
5 years and above	111 (21.18)
**Car ownership, n (%)**	
Owned	159 (30.34)
Rent-to-own	135 (25.76)
Rent	230 (43.90)
**Sleep duration, n (%)**	
6 hours and below	116 (22.14)
6–8 hours	320 (61.07)
8 hours and above	88 (16.79)
**Nap habit, n (%)**	
No nap or less than 10 min	185 (35.31)
Within 30 min	157 (29.96)
Over 30 min	182 (34.73)

Supplemental table 2: Sleep-related and psychological characteristics of the respondents grouped by debt status (N = 524)

	**Debt-free (N=207)**	**With Debt (N=317)**	**Statistics**	**P value**
	**n (%)**	**n (%)**	**Chi-Square**	
**Sleep duration**				
6 hours and below	32 (15.46)	84 (26.50)		
6–8 hours	125 (60.39)	195 (61.51)	17.960	**<0.001**
8 hours and above	50 (24.15)	38 (11.99)		
**Nap habit**				
No nap or less than 10 min	70 (33.82)	115 (36.28)		
Within 30 min	56 (27.05)	101 (31.86)	3.086	0.214
Over 30 min	81 (39.13)	101 (31.86)		
	**Mean (SD)**	**Mean (SD)**	**T-test**	
**Psychological stress[a]**	10.0 (5.03)	13.7 (6.84)	7.020	**<0.001**
**Family-work conflict[b]**	23.3 (8.67)	27.5 (8.20)	5.581	**<0.001**
**Insomnia symptoms[c]**	11.5 (5.78)	15.1 (7.37)	6.269	**<0.001**

[a] Measured by Kessler-6; [b] Measured by Work-Family Conflict Scale; [c] Measured by Insomnia Severity Index.

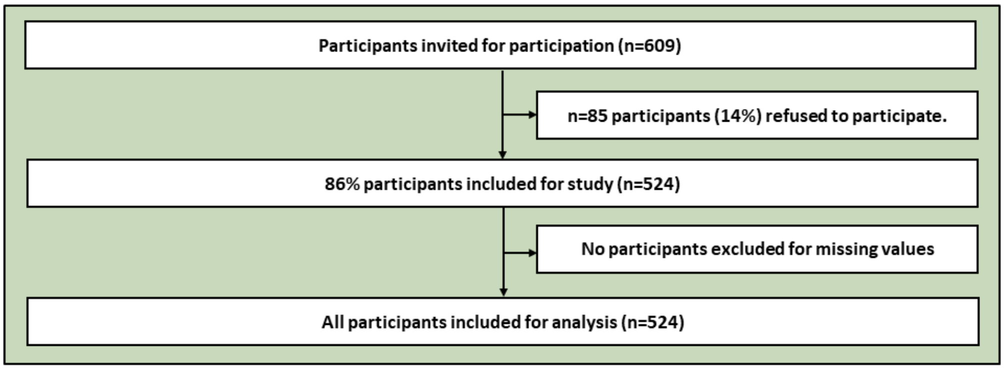

Supplemental figure 1：Flowchart of study participants inclusion.

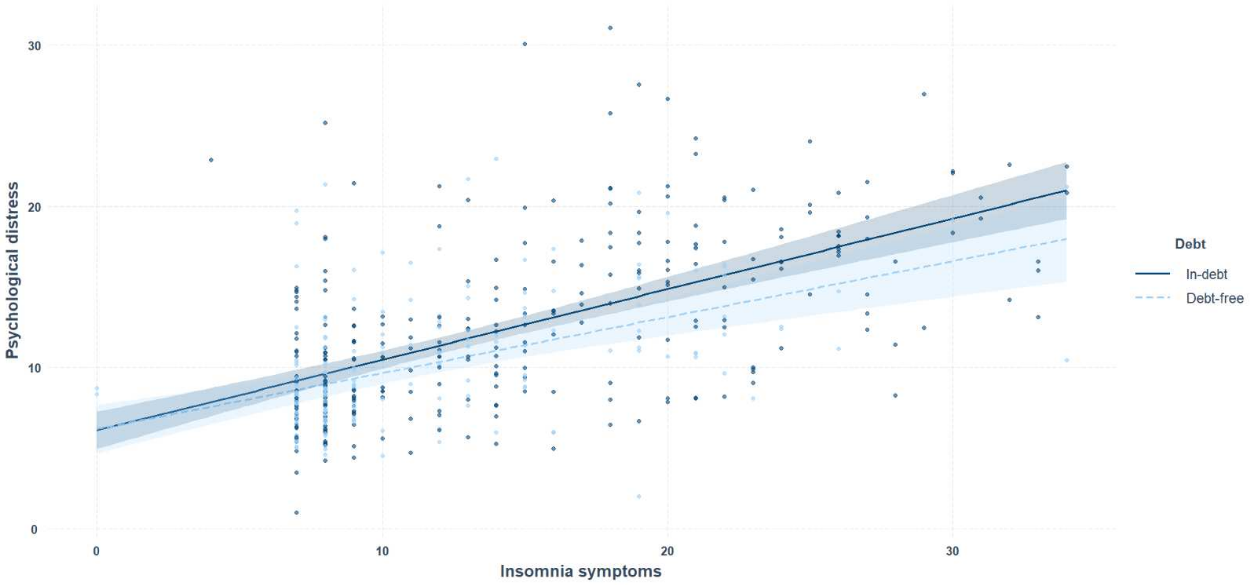

Supplemental figure 2：Scatter linear regression plot describing associations between psychological distress and insomnia symptoms as a function of debt.

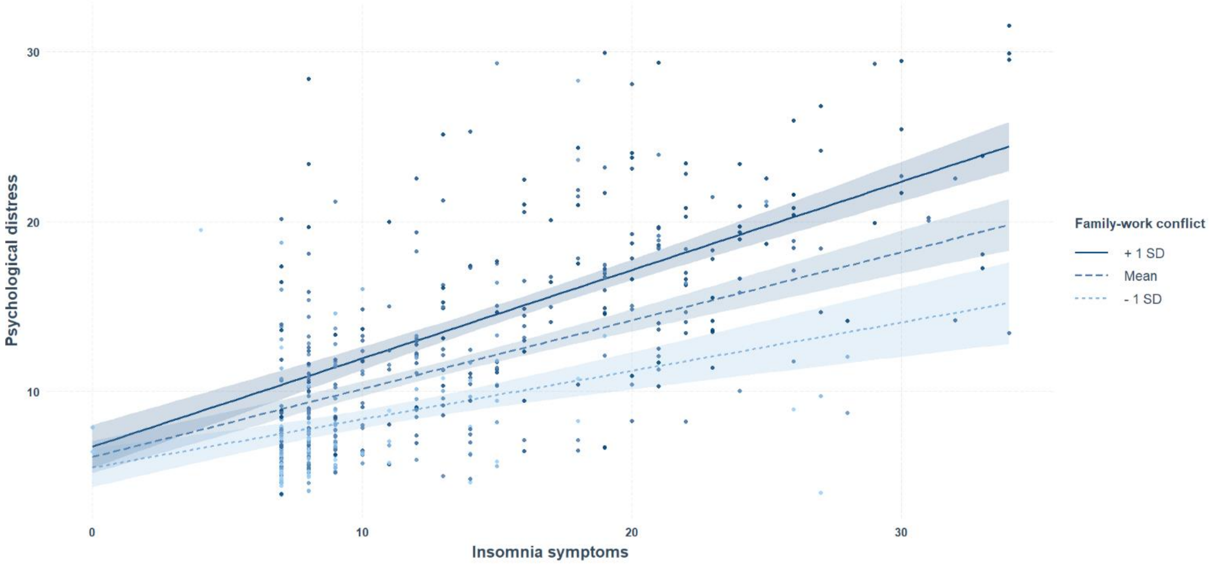

Supplemental figure 3: Scatter linear regression plot describing associations between psychological distress and insomnia symptoms as a function of family-work conflict.
